# Correction: Ahmad et al. Formulation and Optimization of Repaglinide Nanoparticles Using Microfluidics for Enhanced Bioavailability and Management of Diabetes. *Biomedicines* 2023, *11*, 1064

**DOI:** 10.3390/biomedicines13112795

**Published:** 2025-11-17

**Authors:** Mubashir Ahmad, Shahzeb Khan, Syed Muhammad Hassan Shah, Muhammad Zahoor, Zahid Hussain, Haya Hussain, Syed Wadood Ali Shah, Riaz Ullah, Amal Alotaibi

**Affiliations:** 1 Department of Pharmacy, University of Malakand, Chakdara 18800, Pakistan; saharnasim2003@yahoo.com; 2 Center for Pharmaceutical Engineering Science, Faculty of Life Sciences, School of Pharmacy and Medical Sciences, University of Bradford, Bradford BD7 1DP, UK; shahzeb_333@hotmail.com; 3 Department of Pharmacy, Sarhad University of Science and Information Technology, Peshawar 18500, Pakistan; hassan.fls@suit.edu.pk; 4 Department of Biochemistry, University of Malakand, Chakdara 18800, Pakistan; mohammadzahoorus@yahoo.com; 5 Department of Pharmaceutics and Pharmaceutical Technology, College of Pharmacy, University of Sharjah, Sharjah 27272, United Arab Emirates; zhussain@sharjah.ac.ae; 6 Research Institute for Medical and Health Sciences, University of Sharjah, Sharjah 27272, United Arab Emirates; 7 Department of Pharmacy, Shaheed Benazir Bhutto University, Sheringal Dir 18000, Pakistan; haya@sbbu.edu.pk; 8 Medicinal Aromatic and Poisonous Plants Research Center, Department of Pharmacognosy, College of Pharmacy, King Saud University, Riyadh 11451, Saudi Arabia; 9 Department of Basic Science, College of Medicine, Princess Nourah bint Abdulrahman University, Riyadh 11671, Saudi Arabia; amaalotaibi@pnu.edu.sa

## Error in Figure

In the original publication, there was an inadvertent labeling mistake in Figure 11A as published [1]. Figure 11A was mistakenly mislabeled as corresponding to the control group. In fact, it is a second image from the Rp-Nc 1 mg-treated group. This image was captured from the same sample as that shown in Figure 11D. As a result, there is a slight overlap in one corner of Figure 11A,D, since both represent the same treatment group. Because we were capturing images from different parts of the organs, unintentional mislabeling occurred. The corrected version of Figure 11 appears below.

The authors state that the scientific conclusions are unaffected. This correction was approved by the Academic Editor. The original publication has also been updated.

## Figures and Tables

**Figure 11:**
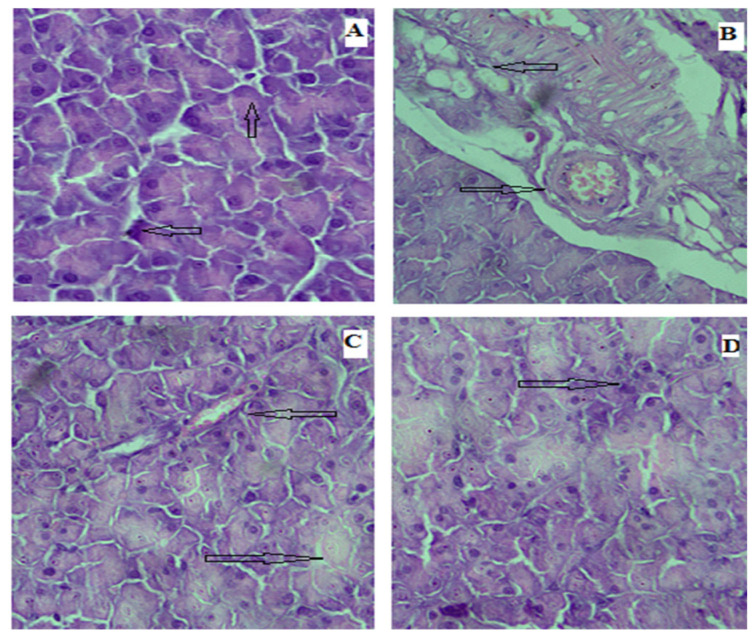
Photomicrographs of microsections of the pancreas of rats: (**A**) control group showing the normal histological structure of islets of Langerhans with normal pancreatic acini; (**B**) diabetic group showing ruptured and destroyed islets of Langerhans with damage in beta cells; and (**C**,**D**) Rp-Nc 0.5 mg- and 1 mg-treated groups showing some normal islets of Langerhans with some mild destruction in normal pancreatic ducts and in between normal pancreatic acini (H&E scale bar, 25 µm).
